# The application of 3D printing technology in multistage functional reconstruction of severe Gustilo IIIb right lower limb injury: artificial hinged knee prosthesis replacement: A case report

**DOI:** 10.1097/MD.0000000000049595

**Published:** 2026-07-03

**Authors:** Wei Zhao, Wujun Xing, Jianruo Zhang, Xiaojun Chen

**Affiliations:** a Department of Orthopedics, Zhenhai District Traditional Chinese Medicine Hospital, Zhenhai District Longsai Medical Group, Ningbo, Zhejiang Province, China; b Department of Orthopedics, Jiamusi Hospital of Traditional Chinese Medicine, Affiliated to Heilongjiang University of Chinese Medicine, Jiamusi, Heilongjiang Province, China.

**Keywords:** 3D printing technology, artificial hinged knee prosthesis, bone and joint reconstruction, Gustilo type IIIb fracture, personalized prosthesis design

## Abstract

**Rationale:**

Gustilo type IIIb fractures are severe open injuries often accompanied by extensive soft-tissue damage, bone defects, and joint destruction, posing significant therapeutic challenges. As an emerging technology, 3D printing offers a novel approach to personalized prosthesis design and bone-joint reconstruction. This case report aims to demonstrate the feasibility and clinical outcomes of applying a staged strategy combined with 3D-printed custom implants to such a complex injury.

**Patient concerns:**

A 31-year-old Asian male sustained a severe open injury to his right lower limb following a traffic accident, presenting with extensive soft-tissue loss, exposed bone, and obvious deformity of the right knee and ankle, accompanied by active bleeding and severe pain.

**Diagnoses:**

Gustilo type IIIb open injury of the right lower limb; destructive injury of the medial knee joint complex; and open fracture of the right medial malleolus.

**Interventions:**

A multistage treatment strategy was employed: multiple planned debridements combined with vacuum sealing drainage and targeted antimicrobial therapy (e.g., linezolid and amikacin); after infection control, fracture treatment with open reduction, internal fixation, and free skin grafting was performed for the ankle. The exposed bone defect on the medial knee was filled with vancomycin-loaded bone paste and covered with a rectus femoris muscle flap transfer. Definitive joint reconstruction surgery was performed 6 months post-injury. Based on mirrored data from the contralateral healthy limb, joint reconstruction was achieved using a rotating-hinge knee prosthesis, restoring the anatomical structure of the segmental femoral and tibial bone defects and the mechanical stability of the knee joint.

**Outcomes:**

The patient recovered well postoperatively, with significant improvement in knee function. The custom augmentation demonstrated close apposition to the host bone with ideal bone healing. Imaging assessment showed satisfactory prosthesis positioning, restored lower-limb alignment and joint-line height, and joint stability, without early prosthesis loosening or other complications. The Hospital for Special Surgery knee score improved from 27 to 83 points, indicating rapid functional rehabilitation.

**Lessons:**

For complex lower-limb trauma with severe bone defects, adhering to the multistage treatment principle combining “damage control” and “functional reconstruction” is crucial. Three-dimensional printing technology enables the customization of prostheses that precisely match individual bone defect morphologies, providing a stable and efficient solution for reconstructing complex bone defects.

## 1. Introduction

Gustilo IIIb open fractures of the lower extremities result from high-energy trauma and are characterized by extensive bone defects, joint instability, and severe soft-tissue damage, significantly increasing the risk of infection, nonunion, and amputation.^[1]^ The core of emergency management is thorough debridement and effective soft-tissue coverage. Muscle flap and local flap transfer techniques are crucial for infection control, improving blood supply, and enhancing limb salvage rates. Special attention must be paid to the frequently associated arterial injuries, as they significantly increase reconstruction complications and are key factors in determining limb salvage strategies and prognosis.^[2]^

Traditional methods of bony reconstruction of the knee joint have limitations. Massive allograft transplantation carries a high risk of complications^[3]^; the Ilizarov bone transport technique, which is capable of addressing long-segment defects, involves prolonged treatment duration and is associated with issues such as pin tract infection and nonunion.^[4]^ Standard hinged knee prostheses often struggle to match complex, individualized bone defect morphologies and have limitations in achieving immediate stability and rapid functional rehabilitation.

Three-dimensional printing technology offers a new strategy for achieving individualized and precise reconstruction. Based on computed tomography (CT)/magnetic resonance imaging data, this technology can be used to design implants that precisely match the complex morphology of bone defects. Their porous structure facilitates bone ingrowth and optimizes mechanical distribution.^[5]^ By integrating these implants with rotating-hinge knee prosthesis systems, this combined approach has the potential to achieve both anatomical reconstruction of the bone defect and mechanical stability of the joint in a single procedure, providing an advanced solution that combines biocompatibility and functionality for treating extremely complex injuries.^[6]^

This report highlights the application of 3D printing technology in the multistage functional reconstruction of an extensive Gustilo IIIb right lower limb injury, particularly through the successful replacement with an artificial hinged knee prosthesis. This demonstrates the potential advantages of 3D printing technology for the treatment of complex fractures. Through retrospective analysis of the patient’s treatment process, postoperative recovery, and imaging results, this study aimed to offer new insights and references for the treatment of similar injuries while further promoting the use of 3D printing technology in orthopedics.

## 2. Case presentation

**Figure 1.**
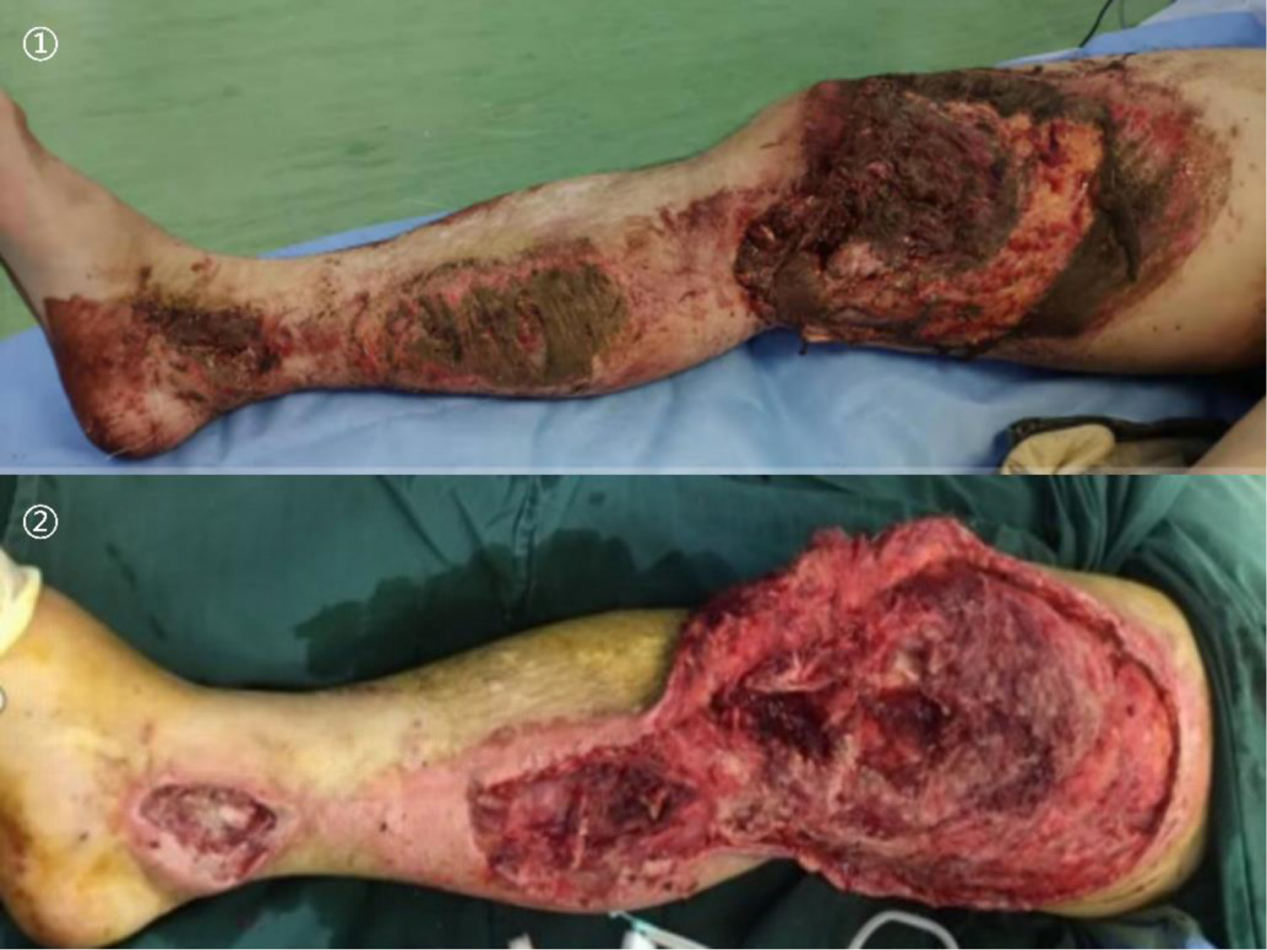
The patient’s condition following initial emergency treatment. Image 1 illustrates the status before debridement, and Image 2 shows the removal of necrotic tissue and contaminants.

The patient was a 31-year-old Asian male farmer with a 13-year history of smoking. He was admitted 4 hours after sustaining a high-energy mechanical impact, resulting in an open, destructive injury of the right knee. The patient’s vital signs were stable. Specialized physical examination revealed a 13 × 18 cm irregular soft-tissue defect on the medial aspect of the right knee, with severely contused wound margins and deep, heavy contamination. The medial joint structures were completely destroyed, with extensive bone loss involving the medial femoral condyle and medial tibial plateau, and exposure of the bone and medullary cavity. The medial collateral ligament and the substance of both the anterior and posterior cruciate ligaments were absent. Distal circulation and sensation in the affected limbs were normal. Diagnosis: Gustilo IIIb, open injury of the right lower limb; destructive injury of the medial knee joint complex (bony and ligamentous structure loss); and an open fracture of the right medial malleolus (Fig. 1).

### 2.1. Treatment strategy

For high-energy open fractures, initial thorough debridement should be completed within 6 to 8 hours post-injury to remove all nonviable tissue and foreign bodies. As a single debridement often fails to fully eliminate concealed necrotic tissue, planned repeat debridements at 24 to 72-hour intervals are crucial until the wound bed appears viable.

Repair of soft-tissue defects is a prerequisite for functional reconstruction. The use of vascularized myocutaneous or fasciocutaneous flaps, which provide both blood supply and coverage, creates conditions for subsequent joint replacement.^[7]^

The management of complex open fractures and joint destruction requires a stepwise principle combining “damage control” and “functional reconstruction.” For ankle fractures, open reduction and internal fixation were performed concurrently with skin grafting on the foot and ankle at the same stage. Considering the patient’s occupation as a manual laborer with high functional demands on the joint, and the “composite tissue” defect accompanying the destructive injury, it was decided to utilize 3D printing to address the missing bone tissue.^[8]^

### 2.2. Treatment course

**Figure 2.**
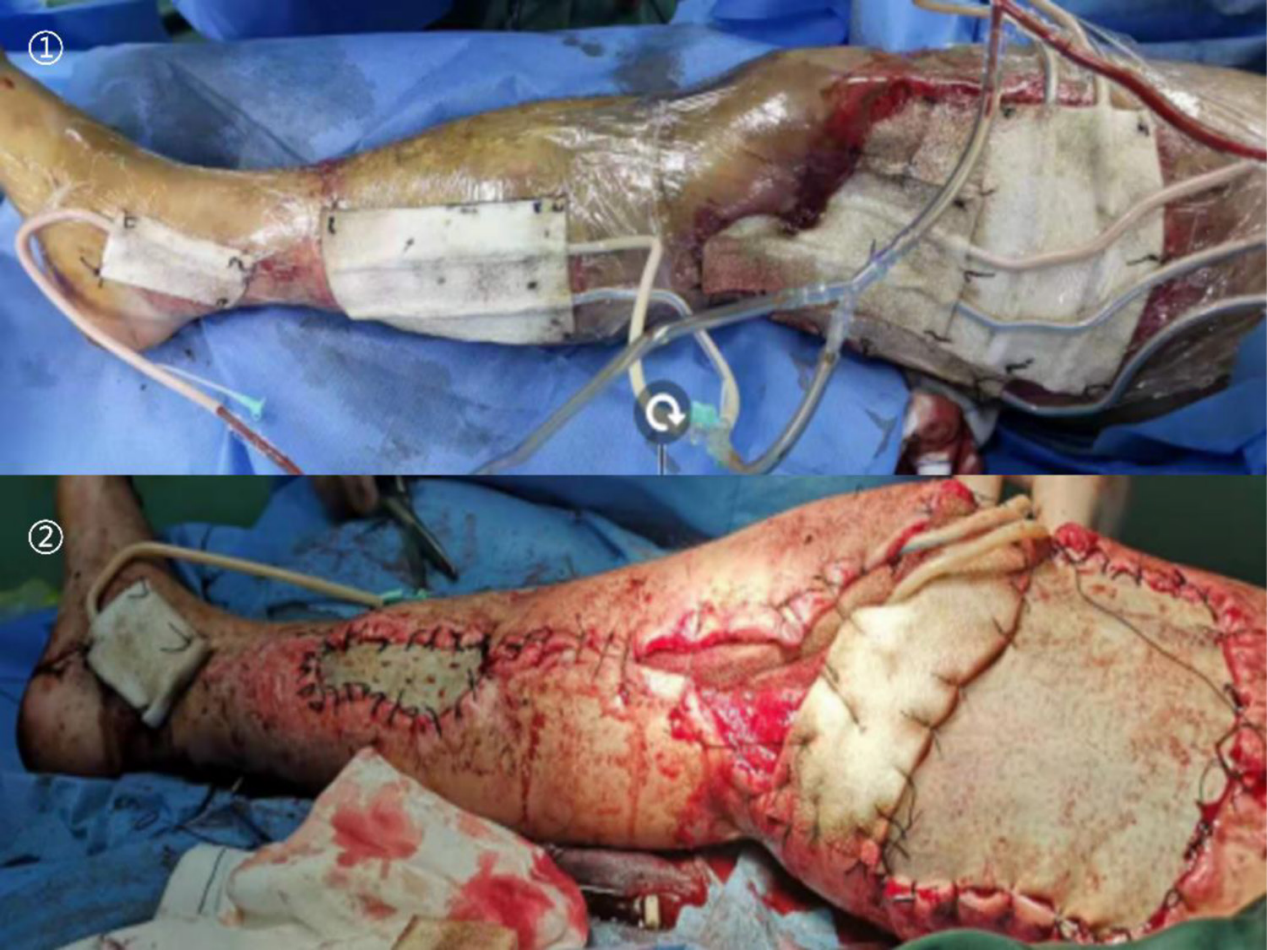
After the thorough removal of contaminants and necrotic tissue, Image 1 shows the application of VSD, and Image 2 shows the free skin graft performed after the formation of granulation tissue. VSD = vacuum sealing drainage.

The patient underwent immediate thorough debridement and vacuum sealing drainage with negative-pressure wound therapy. Empirical second-generation cephalosporin therapy was initiated after initial debridement. Subsequent microbial culture indicated *Staphylococcus aureus* (++++), and a significantly elevated white blood cell count (31.0 × 10^9^/L). Antibiotic therapy was then adjusted to linezolid based on the susceptibility results, and silver ion dressings were applied to the wound for antimicrobial management. Following treatment, the white blood cell count decreased to 16.3 × 10^9^/L, but infection control remained suboptimal. A second debridement was performed, from which *Enterobacter cloacae* (+++) was cultured. Antibiotic therapy was switched to amikacin, which stabilized the infection. This was followed by a third thorough debridement to completely excise the infected and necrotic tissues. Once the wound bed displayed healthy granulation tissue, open reduction and internal fixation of the ankle fracture were performed, supplemented with a free skin graft for coverage. For the area of exposed bone on the medial aspect of the knee, vancomycin bone marrow paste was used to fill the bone defect, and coverage was completed using a rectus femoris muscle flap transfer (Fig. 2).

### 2.3. Joint reconstruction surgery

Joint reconstruction was performed 6 months after complete infection control. The surgery utilized the Zimmer NexGen rotating-hinge knee prosthesis system combined with a customized titanium alloy augment fabricated based on 3-dimensional reconstruction and computer-aided design to reconstruct segmental bone defects of the femur and tibia and restore knee joint mechanical stability.

#### 2.3.1. Preoperative planning and implant design

**Figure 3.**
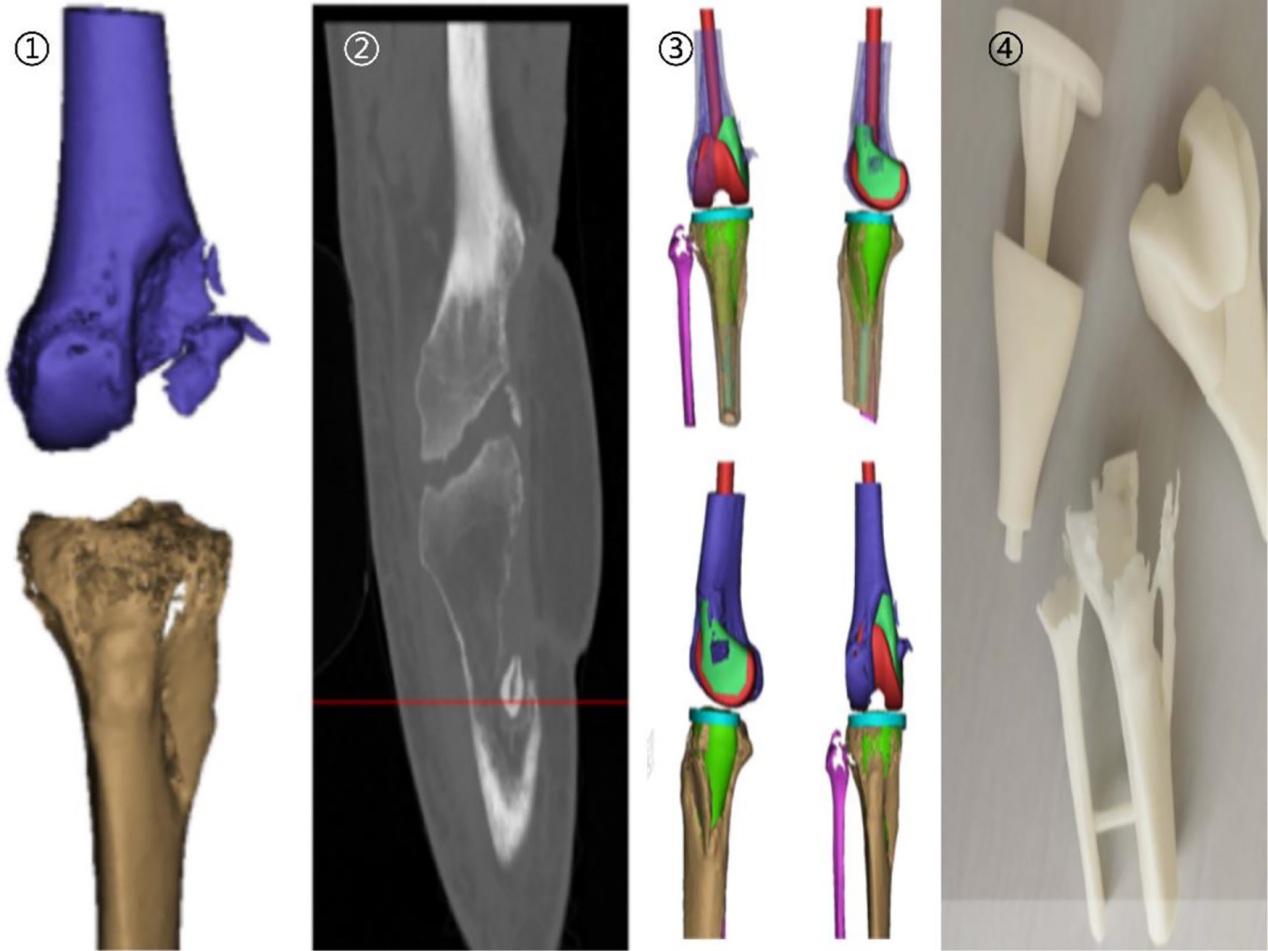
The patient underwent CT scanning and 3D reconstruction (Images 1 and 2), and 3D printing scans and models were created from the imaging data on the computer (Images 3 and 4). CT = computed tomography.

Based on digital imaging and communications in medicine data obtained from CT scans of the contralateral healthy lower limb, a 3-dimensional model of the knee joint was reconstructed using Mimics software (version 21.0; Materialise’s interactive medical image control system) and mirrored to simulate normal lower-limb alignment and joint morphology. Customized titanium alloy augmentation was designed for the femoral and tibial defect areas. Its bone-contacting surface features a porous structure (porosity 60%–70%, pore size 500–600 μm) to promote osseointegration. The augment was designed with a mechanical interface compatible with the NexGen system and manufactured using selective laser melting technology followed by sterilization (Fig. 3).

#### 2.3.2. Surgical procedure

Osteotomy and bone bed preparation: Osteotomy was guided by patient-specific cutting guides based on 3-dimensional data. Referencing the 3D-printed positioning guide method, a reference plane 8 to 9 mm below the tibial plateau with a thickness of 3 mm was established. The tibial rotational axis was determined by projecting onto the femoral transepicondylar axis. First, a tibial prosthetic platform was developed. The intramedullary canal was progressively reamed, starting from a diameter of 9 mm, to ensure that the osteotomy surface was perpendicular to the mechanical axis. Subsequently, the femoral medullary canal was prepared and progressively reamed to accommodate the femoral stem base and stem extension.

#### 2.3.3. Defect reconstruction and trial reduction

The customized titanium alloy augmentation was installed to cover the bone defect areas. Tibial and femoral trial components, along with a temporary articular surface, were sequentially implanted. Following the principles of flexion and extension gap balance, lower-limb alignment, joint-line height, and soft-tissue tension were assessed intraoperatively. Patellar tracking was confirmed to be normal without tilt or subluxation.

#### 2.3.4. Prosthesis fixation and assembly

**Figure 4.**
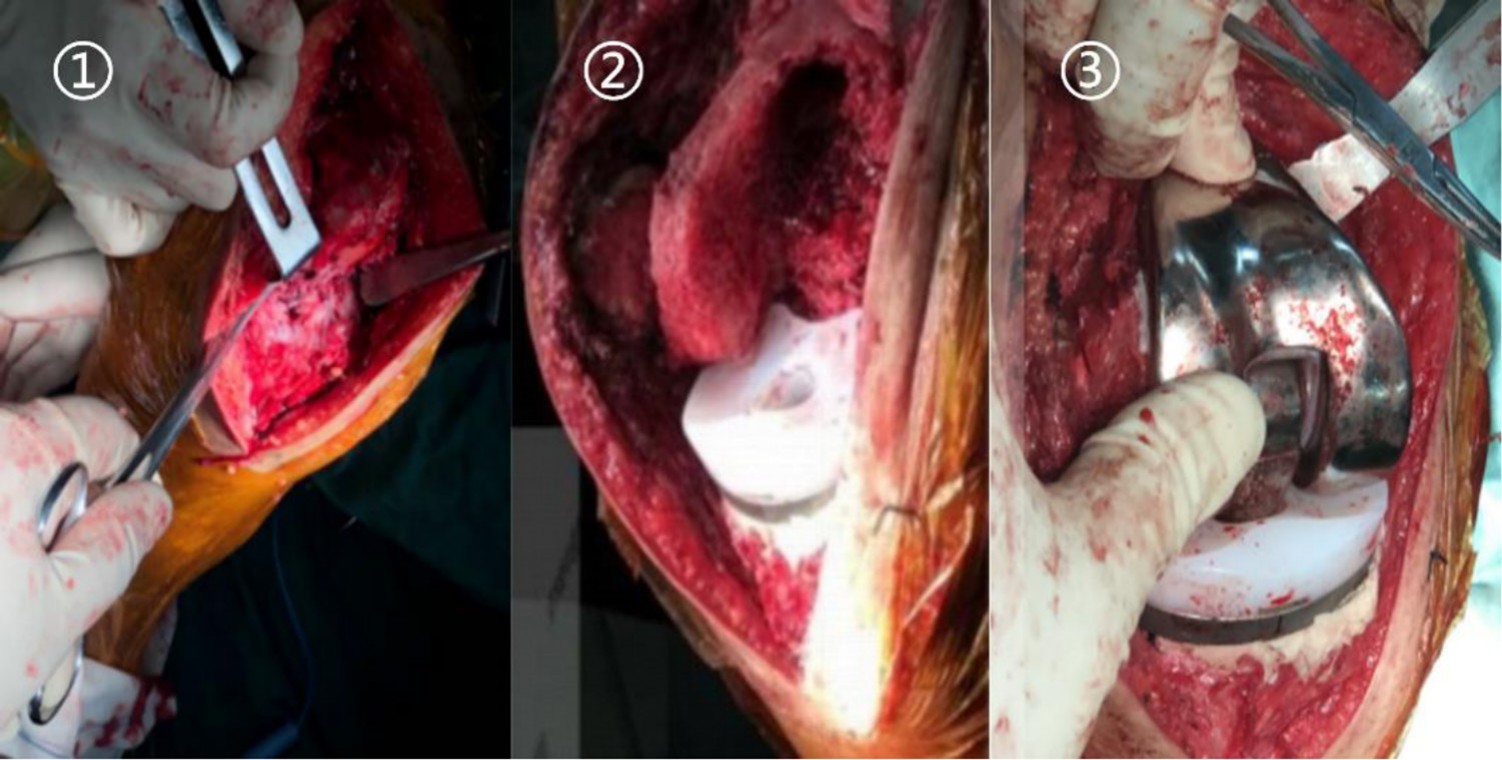
The surgical procedure during joint reconstruction: Image 1 depicts osteotomy, and Images 2 and 3 illustrate the installation of the artificial joint.

A staged cementing technique was used. First, the tibial baseplate component was fixed using vancomycin-loaded bone cement and cured. Subsequently, the femoral condylar component and the tibial tray were cemented. During this step, the hinge post was rotated anteriorly to prevent cement intrusion into the articulation. Finally, the polyethylene bearing was inserted, and the hinge post extension was placed and locked to a torque of 15 N·m (Fig. 4).

#### 2.3.5. Postoperative assessment

Intraoperative blood loss during the final reconstruction surgery was 200 mL. Postoperative imaging showed satisfactory prosthesis positioning, restored lower-limb alignment, reconstructed joint-line height, and a stable knee in both flexion and extension. The customized augmentation demonstrated close apposition to the host bone, providing a structural foundation for osseointegration.

### 2.4. Results

**Figure 5.**
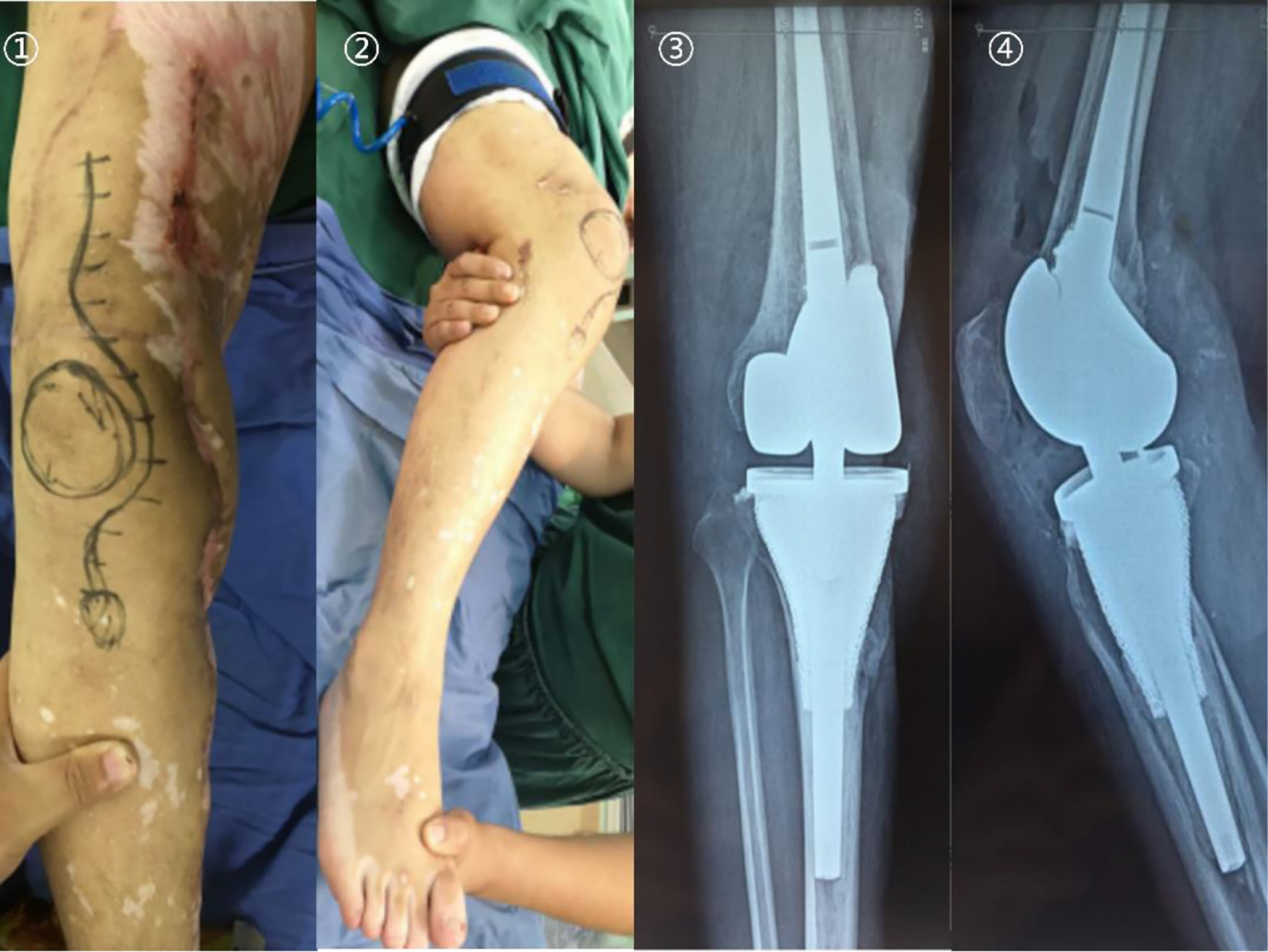
The preoperative status of the patient’s joint replacement is shown in Images 1 and 2, depicting the condition approximately 6 months after skin flap surgery. Images 3 and 4 represent the postoperative anteroposterior and lateral views following joint reconstruction.

The perioperative data indicated that the patient’s hospital stay for joint reconstruction surgery totaled 20 days, with a postoperative hospitalization period of 17 days. Second-generation prophylactic cephalosporins were administered. No complications, such as deep vein thrombosis, flap necrosis, or early prosthesis loosening, occurred. The patient began standing with weight bearing on the third postoperative day. Postoperative radiographs showed satisfactory positioning of the prosthesis. Assisted walking with a brace commenced on the fifth postoperative day, and the patient was discharged on postoperative day 14 after suture removal. Full weight-bearing walking was achieved at the 6-week (42-day) follow-up visit. Serial postoperative Hospital for Special Surgery knee scores, assessed weekly starting from the seventh postoperative day, were 27, 61, 81, 76, 84, and 83 points, respectively, demonstrating significant functional improvement (Fig. 5).

## 3. Discussion

### 3.1. Decision-making regarding the timing of reconstruction

For Gustilo IIIb fractures, the traditional management principle emphasizes emergency thorough debridement within 6 to 8 hours post-injury to minimize the risk of infection. Recent evidence-based medicine suggests that surgical timing can demonstrate greater flexibility in specific situations, such as when patients present with severe polytrauma or under resource-limited conditions. Studies indicate that with timely and effective systemic antibiotic coverage (e.g., second-generation cephalosporins), extending the initial debridement window to within 24 hours does not significantly increase the infection rate.^[9]^ In such cases, treatment decisions should be based on a comprehensive assessment of the patient’s overall condition, the extent of soft-tissue damage, and the infection risk.^[10]^

The timing of definitive bone and joint reconstruction in subsequent stages requires greater caution. Although aggressive single-stage reconstruction can shorten the overall treatment course, it requires a clean wound bed and complete infection control.^[11]^ For patients with compromised soft-tissue conditions or ongoing infection risk, a staged strategy is more prudent. Initially, an antibiotic-loaded bone cement spacer is used to maintain the bone defect space and control infection. Definitive reconstruction is performed only after inflammatory markers have normalized and soft-tissue healing is stable. This “delayed-definitive” strategy allows more ample time for preoperative planning and prosthesis fabrication and can reduce the risk of postoperative deep infection.^[12]^

### 3.2. Strategy for precision antimicrobial therapy

Infection control is the key determinant of limb salvage and reconstruction success in managing severe open injuries, such as Gustilo IIIb fractures. For infections commonly associated with trauma, such as those involving methicillin-resistant *S aureus*, linezolid has significant advantages. As an oxazolidinone antibiotic, it exhibits potent activity against gram-positive bacteria, particularly methicillin-resistant *S aureus*.^[10]^ Multiple studies have confirmed that linezolid has superior clinical and microbiological success rates with a significantly lower risk of nephrotoxicity. The similar bioavailability of oral and intravenous formulations facilitates sequential therapy, shortens hospital stays, and offers pharmacoeconomic benefits. Combination regimens with fosfomycin provide a new synergistic treatment strategy for complex infections.^[13]^

At the local infection control level, silver-coated surfaces are often applied to implants or wound dressings to provide broad-spectrum antibacterial protection through sustained silver ion release, with potential value in inhibiting biofilm formation.^[14]^ This approach is particularly suitable for soft-tissue defect areas with poor blood supply and serves as an adjunct to systemic antibiotics. However, its efficacy is not absolute. For instance, some *E cloacae* strains have been confirmed to carry silver resistance genes, which may compromise the effectiveness of the coating. Therefore, silver coatings should be viewed as part of a comprehensive anti-infection strategy, and their application requires prudent evaluation based on specific etiological evidence and the wound environment.^[15]^

### 3.3. Weighing reconstruction pathways

For devastating knee joint injuries accompanied by severe bone defects, treatment options require careful weighing between bone regeneration potential and functional recovery efficiency. The Ilizarov technique enables true biological bone regeneration through bone transport, avoiding the risks associated with allogeneic materials; however, its treatment cycle is prolonged, and the significant discomfort and nursing burden imposed by the external fixator limit its application.^[16]^ By contrast, 3D printing offers an innovative solution for rapid functional reconstruction. Custom-manufactured porous metal cones or augments can not only precisely match complex bone defects and provide excellent axial and rotational stability^[17]^ but also optimize surgical outcomes through seamless integrated interface design.^[18]^ Traditional methods, such as bone cement, metal augments, and structural allografts, often pose challenges in managing severe bone defects encountered in revision surgeries, whereas 3D printing technology provides an innovative solution that enables structural reconstruction without significantly increasing the risk of infection.^[19,20]^

The selection of a treatment plan must be deeply integrated with the patient’s age and physiological status. Younger patients, typically those with better bone density and cartilage quality, are more suitable for aggressive surgical repair. For example, a combined approach of high tibial osteotomy and osteochondral autograft transplantation has demonstrated good long-term survival rates and functional improvement in young, active patients.^[21]^ In contrast, elderly patients, who often present with osteoporosis, diminished cartilage self-repair capacity, and higher surgical risks, require more cautious strategies.^[22,23]^ Studies suggest that bone density and cartilage volume are important predictors of cartilage degeneration and are key factors in treatment decision-making for elderly patients.^[24]^

In summary, decision-making should be based on a comprehensive assessment of defect characteristics, patient age, bone and cartilage condition, functional expectations, and risk tolerance. The Ilizarov technique is suitable for patients who prioritize biological regeneration and can tolerate long-term treatment, whereas 3D printing technology offers a reliable option for complex cases pursuing anatomical reconstruction and rapid functional recovery. For elderly patients, a balance must be sought between surgical intervention and conservative management, or auxiliary measures, such as bone density enhancement, should be integrated to optimize prognosis.

### 3.4. Key technical points for precision reconstruction and implant considerations

When performing reconstruction surgery with a rotating-hinge knee prosthesis, the precision of surgical execution and the rationality of implant design form the cornerstone of long-term outcomes. The core of achieving anatomical reconstruction is precise osteotomy and alignment control.

Accurate osteotomy is the foundation for reconstructing normal lower-limb alignment, joint-line height, and prosthesis rotational positioning. Custom 3D-printed osteotomy guides based on CT data from the contralateral healthy limb can assist the surgeon in achieving precise matching between osteotomy surfaces and custom augments, thereby systematically optimizing these parameters.^[25]^ However, the application of this technology requires careful consideration of individual variations. When the degree of degeneration differs between the 2 knees or when congenital deformities exist, simply mirroring data from the healthy side may not be appropriate, necessitating comprehensive intraoperative assessment.^[26]^ Although modern computer navigation technology can provide high-precision guidance for the osteotomy plane, manual operation during the final prosthesis implantation and cement fixation phases may still introduce minor alignment errors.^[27]^ Therefore, meticulous verification of the final alignment is essential before the cement hardens.^[28]^ Regarding specific parameters, intraoperative attention should be paid to restoring the physiologically appropriate tibial posterior slope. Studies have suggested an average of approximately 11° in Asian populations, which can serve as a crucial reference.^[29]^ Concurrently, the rotational alignment of the tibial component must be strictly controlled to avoid excessive internal rotation, as this has been shown to significantly increase articular contact forces and induce abnormal femoral rollback, compromising joint stability.^[30]^

The material and structural properties of an implant directly influence its long-term mechanical stability, risk of subsidence, and aseptic loosening. The key to the current design is to promote osseointegration and optimize load transfer. Employing a porous structure with specific parameters at the bone-implant interface can effectively guide bone ingrowth, achieving reliable biological fixation and thereby significantly reducing the incidence of aseptic loosening. The rotating-hinge mechanism provides inherent stability in both the coronal and sagittal planes of the knee joint, making it particularly suitable for complex cases involving extensive post-traumatic ligamentous insufficiency. Furthermore, it is critical to focus on the long-term wear resistance of prostheses. Optimizing material pairing and manufacturing processes, such as using highly polished tibial tray surfaces, can effectively reduce the backside wear of polyethylene inserts, which is of significant value in extending the service life.^[31]^

Ultimately, the success of surgery is reflected not only in the precision of radiographic alignment but also in its translation into favorable clinical function. Research has indicated a significant correlation between postoperative knee kinematics and patient-reported functional outcomes.^[32]^ Therefore, by integrating the aforementioned precise surgical techniques, rational prosthesis alignment, and advanced design concepts, the aim is to restore near-physiological joint kinematics, maximally enhance postoperative joint stability and functional recovery, and effectively control the long-term risks of prosthesis subsidence and revision.

## 4. Conclusion

This patient underwent multistage treatment and functional reconstruction, resulting in partial recovery of right lower-limb function. Throughout the treatment process, thorough debridement, infection control, wound repair, and functional reconstruction were all crucial steps. For similar cases, a personalized treatment plan should be formulated based on the patient’s specific condition and the severity of the injury, with an emphasis on mastering the surgical techniques and key technical principles.

## Acknowledgments

We would like to express our sincere gratitude to the teams of Director Zhang Jianruo and Director Xing Wujun for their invaluable contributions to this study. We extend special thanks to Director Zhang Jianruo and Director Xing Wujun for their guidance in clinical case collection, treatment planning, and implementation.

We are also grateful to all the medical staff at Zhenhai Traditional Chinese Medicine Hospital and Jiamusi Traditional Chinese Medicine Hospital for their dedicated work and assistance, particularly in data collection and patient management.

Finally, we thank the patients and their families who participated in this study for their cooperation and trust throughout the treatment process. Without their support, this study would not have been possible.

## Author contributions

**Investigation:** Wei Zhao.

**Project administration:** Wei Zhao.

**Methodology:** Wujun Xing.

**Supervision:** Wujun Xing.

**Writing – original draft:** Wei Zhao, Xiaojun Chen.

**Writing – review & editing:** Wei Zhao, Jianruo Zhang.
